# Three Cases of Emergency Department Medical Malpractice Involving “Consultations”: How Is Liability Legally Determined?

**DOI:** 10.5811/cpcem.2021.7.52680

**Published:** 2021-08-04

**Authors:** Alaa Aldalati, Venkatesh R. Bellamkonda, Gregory P. Moore, Alexander S. Finch

**Affiliations:** * Mayo Clinic College of Medicine and Science, Mayo Clinic School of Graduate Medical Education, Rochester, Minnesota; † Mayo Clinic College of Medicine and Science, Department of Emergency Medicine, Rochester, Minnesota

## Abstract

This article presents three successfully litigated medical malpractice cases involving emergency physicians and consultants. We discuss the respective case medical diagnoses, as well as established legal principles that determine in a court proceeding which provider will be liable. Specifically, we explain the legal principles of “patient physician relationship” and “affirmative act.”

## INTRODUCTION

Emergency physicians (EP) often consult other specialty services to assist in optimal patient care. Here we present three cases that involved consultation and ultimately resulted in successfully litigated malpractice cases. Many EPs and consultants are unaware of the legal principles that govern their liability if a lawsuit is pursued. Specifically, we clarify what defines a “patient physician relationship” and an “affirmative act” as well as the pathophysiology and risk management of the diagnosis in each case.

### Case 1: Sozomentou v Arfaras – Florida

A 66-year-old man presented to an emergency department (ED) with chest pain. After examination by the EP he was evaluated with a chest radiograph. The EP interpreted the study as “within normal limits” and reviewed the radiologist’s interpretation of “top normal size heart with tortuous aorta.” The patient was subsequently admitted to the hospital with a diagnosis of chest pain and rule out myocardial infarction. That evening, he died from an aortic dissection (AD) with associated pericardial tamponade.

A lawsuit brought against the EP and the radiologist claimed that the radiologist failed to recognize and suggest to the EP the possibility of an AD and the need for immediate computed tomography (CT) of the chest. A second claim alleged that the EP failed to include AD in the differential diagnosis and order a CT of the chest. The radiologist maintained that they did not exclude the diagnosis and it is the EP’s duty to clinically provide appropriate care and orders. After deliberation, a jury awarded the plaintiffs $6.4 million; they assigned 2% of the amount to the radiologist, 80% to the EP, and 18% to the inpatient provider.^1^

### Case 2: Estate of Fischel v Mujic M – New Jersey

A 39-year-old man presented to the ED via ambulance complaining of difficulty breathing. An electrocardiogram (ECG) was done. The cardiologist who over-read the tracing reported findings consistent with myocardial infarction and communicated this with the EP. The EP discharged the patient with a diagnosis of bronchitis. The patient’s wife had delivered a son two days prior and was discharged from the hospital at the same time. The next day the man collapsed on the floor and cardiopulmonary resuscitation was initiated. An ambulance was called, but nevertheless he died.

A lawsuit was brought claiming that the EP should not have discharged the patient, especially with the cardiologist’s interpretation of the ECG obtained in the ED. The cardiologist claimed the entire fault rested with the EP. The EP’s defense was that his care was reasonable and he was not informed by the cardiologist of his reading. The case was settled by the EP for $2 million.^2^

### Case 3: Anonymous N.P. v Anonymous Physician

Parents brought their three-year-old son to the ED complaining that he had put a watch battery in his nose. The provider did radiographs of the chest and abdomen and did not see a foreign body. On nasal examination they were unable to visualize the battery. An otolaryngologist (ENT) was called; the emergency provider was instructed to have the parents call the ENT office at eight in the morning for their son to be seen, and to have the child not take any oral liquids or solids after midnight and wait until he was seen by the ENT physician. The parents asked if their child could be taken to a nearby children’s hospital, but they were reassured that it was not necessary and were also instructed to return if there was any respiratory distress.

About six hours later the child began having discharge from his nose. His parents took him to another hospital where a specialist removed the watch battery from his nose. By that time there was extensive tissue necrosis resulting in a perforated nasal septum requiring surgery. A lawsuit for malpractice was filed with the court for delay in care. The lawsuit was filed against the emergency provider and the ENT specialist. The ENT physician asked for the case to be dismissed with respect to his care, claiming he did not have a physician-patient relationship. He also claimed that the plan was a general one in the event he did see the patient the next day and that it was not specific care.

A physician-patient relationship is required as the first element of a malpractice action. The court stated: “there is no physician patient relationship if the physician does not see, treat, or in any way participate in the care and diagnosis.” The court also said “the physician must perform some affirmative act.” The court examined the facts and declared that the ENT physician had placed an order for “nothing per mouth” status and directed when follow-up care was required, demonstrating a participation in care that constituted an affirmative act.^3^

## DISCUSSION

### Aortic Dissection and Risk Management

Acute AD is rare, extremely dangerous, and presents variably, resulting in a diagnostically challenging and high-risk situation for EPs. Aortic dissection has an estimated incidence of about 0.006% per year.^4^ Despite the rarity of AD, mortality may reach as high as 50% at 48 hours, 90% at one month if not operated upon, and even 30% at one month if operated upon.^5^,^6^

As a possible diagnosis, AD may occur secondary to predisposing factors including hypertension and smoking, as well as genetic conditions such as Marfan syndrome, congenital bicuspid aortic valve disease, and vasculitides. Aortic dissection may be acquired secondary to trauma or iatrogenically from healthcare procedures.^7^ While chest pain is the most frequent symptom reported in patients with acute AD, back pain, abdominal pain, syncope, neurologic symptoms, hypoperfusion syndromes, and other symptoms have been reported as well.^8^

Ordering the appropriate testing to evaluate for dissection is critical. Intravenous contrast-enhanced CT has sensitivity and specificity values between 95–98% and is widely available compared with transesophageal echocardiography and magnetic resonance imaging, making CT the mainstay of evaluation in EDs nationally.^8^ Evaluation of chest pain, which is the presenting complaint for about 10 million ED visits annually,^9^ costs the healthcare system about $10 billion each year in the United States.^10^ Indiscriminate use of CT may pose a risk from radiation to individual patients, as well as unnecessary cost. To assist the clinician in making a determnation, many have incorporated the use of D-dimer to exclude the diagnosis, citing its very high sensitivity values.^11^ However, a clinical policy statement from the American College of Emergency Physicians (ACEP) discourages reliance on D-dimer as a singular tool to exclude the diagnosis because the quality of studies supporting this practice are felt to be inadequate (February 2018). The European Society of Cardiology recommends using D-dimer to shift the degree of suspicion up and down rather than to make or exclude the diagnosis.^4^

When examining litigation of AD cases, a review by Elefteriades et al included 23 patients who had acute ADs, 22 of them fatal. The most common category of malpractice alleged was failure to diagnose or delayed diagnosis. This review showed that lawsuits were brought against a variety of physicians including EPs, radiologists, cardiothoracic surgeons, and many others including obstetrician-gynecologists. In about two thirds of the cases, the medical care was felt to be suboptimal. The authors recommended simply including AD in the differential diagnosis, performing appropriate testing to assess the likelihood of this disease, and interpreting the results of the testing correctly. These three steps will both enhance diagnosis and decrease the likelihood that patients and families will perceive that they were treated suboptimally, reducing the chance of litigation.^12^

### Electrocardiograms and the Diagnosis of Acute Myocardial Infarction

The American Heart Association estimates that a myocardial infarction will occur approximately every 40 seconds in the US, making it a common emergency faced by healthcare teams. Unfortunately, heart disease is also the leading cause of death, representing just over 840,000 deaths domestically in 2016.^13^ Given the significant morbidity and mortality, this disease process is a focus of emergency medicine (EM) practice. Screening ECGs are commonly performed and read for findings associated with acute myocardial infarction (AMI). Acute coronary syndrome (ACS), as a spectrum of disease, is commonly associated with chest pain; however, dyspnea, diaphoresis, and jaw and arm pain are also presenting symptoms.^14^

Electrocardiogram changes can raise concern for AMI and ischemia. Furthermore, in many settings ECG findings in conjunction with a concerning history are an indication for percutaneous intervention (PCI) or thrombolytics with a goal of restoring myocardial perfusion. Well established benchmarks of door-to-intervention times for both thrombolytics and PCI highlight the principle that any delay in diagnosis or treatment of AMI is deleterious to the patient.^15^ This principle was underscored by ACEP in its updated 2017 clinical policy statement “ED Management of Patients Needing Reperfusion Therapy for Acute ST-Segment Elevation Myocardial Infarction,” where there was a Level B recommendation that fibrinolytics be administered to patients when door-to-balloon time is anticipated to exceed 120 minutes.^16^ These well delineated benchmarks outline an expected standard of care for a high-risk disease process, which can also factor into the medicolegal risk involved.

Patients with missed AMI on their index visit are at increased risk for ensuing cardiac events.^17^ These adverse outcomes increase morbidity and mortality for this patient population.^18^ Thus, careful interpretation of a patient’s ECG by the EP in conjunction with a detailed history and physical exam are necessary in all cases of patients presenting with symptoms that may be associated with AMI. In assessing the literature evaluating the medicolegal consequences of missed myocardial infarction, two clear themes emerge: 1) ACS represents a high-risk disease entity that is associated with more malpractice dollars recovered than any other condition^19^; and 2) it is a common source of litigation in the ED, representing up to 20% of EM-associated settlement funds.^17^,^20^

A 2010 review of ED malpractice claims found that AMI was the second most common disease process associated with a claim (5% of claims).^21^,^22^ Recognition of AMI based on ECG findings can be difficult and prone to error, making these misinterpretations among the costliest mistakes in terms of malpractice dollars.^23^ Actual rates of missed AMI are unknown, but studies estimate this to be about 2% in the US, which is quite high for such a high-stakes diagnosis.^18^,^19^ A more recent article affirmed that AMI accounts for the second most common malpractice claim in ED and urgent care settings.^24^

Translating an ECG into an AMI diagnosis requires both accurate interpretation of the ECG and correct extrapolation that the visualized pattern represents disease and not a mimic of disease. Studies looking specifically at ECG interpretations have found that misdiagnosis rates and accuracy can be variable among EPs. One study that focused on ECGs with ST-segment elevation found there was misinterpretation as to the underlying cause of the elevation in 5.9% of cases.^25^ Another study found that accuracy in identifying ST-segment elevation myocardial infarction (STEMI) representing acute coronary occlusion based on ECG was only 69.1%.^26^ Finally, these themes exist not only in the US but are seen internationally as well. A 2017 study of closed malpractice claims associated with AMI in Taiwan found that misdiagnosis was the most common dispute associated with claims.^27^

In summary, ACS represents a disease process with increased morbidity and mortality to the patient if the diagnosis is missed. It is a relatively common source of litigation with high malpractice recovery and therefore represents a significant medicolegal risk. Given this risk, great care must be taken to consider ACS in the differential and to evaluate it thoroughly.

### Nasal Button Battery Foreign Body

While most EPs are aware of the morbidity associated with gastrointestinal ingestion of button batteries, many may not be familiar with the significant risk when they are located in the nose. Patients are often asymptomatic, and many cases are unanticipated discoveries. Nasal foreign bodies (FB) are usually lodged in the floor of the nasal passage, below the inferior turbinate, or in the superior fossa anterior to the middle turbinate. Patients typically present with one-sided, foul-smelling nasal discharge. Having patients simply blow their nose while closing the alternate side may remove the FB. If it is not visualized, then referral to a specialist is indicated.

The vast majority of patients in these cases are less than eight.years old. Button batteries are FBs that release toxic substances (eg, silver, zinc, mercury, or lithium), and cause local electrical burns. An electrical circuit is completed when lodged, and injury usually happens on the anode side of the battery. Subsequent electrolyte leakage results in a caustic injury. Pressure necrosis caused by an impacted FB is a third mechanism of pathology. The complications in the nasal cavity are based on length of time between placement and removal, the orientation of the impacted battery, and the site of contact of the negative pole (anode). If the anode is in contact with the septum or turbinate, then ulceration can occur in just 3–6 hours. Ulceration of the inferior meatus, saddle deformities, chondritis, rhinitis, and alar collapse can eventually result as well. The EP must obtain emergency removal when encountering a button battery in a patient’s nose.^28^,^29^

### Legal Concepts when Determining Liability Regarding ED Consultants

To successfully litigate a malpractice claim, four legal elements must be met: 1) a duty was owed to the patient; 2) the duty was breached (standard of care not met); 3) injury occurred to the patient; and 4) the injury was caused by the breach of duty (causation). To prevail in a malpractice lawsuit against a physician, the first element of a patient-physician relationship (duty) must be established. While an in-person evaluation may satisfy this element, it is clear in the case law that the relationship actually hinges on whether an affirmative act was performed by the physician on behalf of the patient. As in our reported pediatric case involving a retained battery demonstrates, the affirmative act can occur even via a telephone call.

Courts have further delineated duty. In *Walters v Rinker*, the court defined an affirmative act by the physician as an action for the benefit of the patient. For example, this would involve participation in diagnosis or treatment of the patient.^30^ In contrast, merely having physical contact with a patient does not establish a physician-patient relationship. In *Giles v Anonymous Physician I*, a hospitalist presented to the bedside of a patient and checked her medical chart. The hospitalist quickly realized that the patient would not be appropriate for their service. The court found that the hospitalist did not perform any affirmative act by presenting to the bedside or reviewing the medical record and, therefore, there was no physician-patient relationship.^31^

Emergency physicians commonly discuss patients with specialists by telephone. In the event of an adverse patient outcome resulting in a malpractice suit, consultants may be liable only if they establish a physician-patient relationship with the plaintiff. Courts have found that this requirement exceeds the scope of many “curbside” conversations but is established only when the specialist takes an affirmative action toward the patient. This often entails participating in diagnosis or treatment, which would be an action for the benefit of the patient.

Establishing a patient-physician relationship sets up the basis to create medical liability on the physician’s part. Two essential parts confirm this relationship: 1) the patient requests, either directly or via representatives, that a physician provide care; and 2) the physician in return performs an “affirmative act” or shows “intent of care.” It is important to keep in mind that the affirmative act is not only applicable after a direct physician-patient contact. For example, if a test was interpreted by a specialist and advice was given based on it, a patient-physician relationship is established.^32^ In *Walters v Rinker*, examining a pathological specimen was enough to establish a patient-physician relationship as the recommendations were used to plan further treatment. Despite an initial pathology report stating that the finding appeared to be benign, two years later the patient was diagnosed with large cell lymphoma in the setting of declining physical health. The court in this case decided that since the treatment and interventions were based on this test result, the pathologist performed an affirmative act to contribute to this patient’s care.^30^

Medicine is a collegial profession. Often, EPs “curbside” other consulting services as “informal” consults regarding test interpretation or further guidance on treatment. For a consultant to be held legally liable usually a patient-physician relationship must be established.^32^ Generally, physicians who are not on duty are not obligated to treat patients; therefore, they mostly are not held accountable.^33^ Moreover, an on-call consultant giving general advice over the phone does not necessarily establish a relationship with the patient regardless of whether the caller was another physician or the patient. The relationship is established by providing patient- specific advice, or giving direct recommendations to the patient directly or through another party such as the EP.^34^

In addition, documenting in the patient’s chart or billing the patient for a consult, even if there was no physical interaction, strongly implies that a patient relationship exists via an affirmative act. Generally, unless one of the aforementioned circumstances are met, courts tend to be hesitant to hold consultants strictly liable. The courts generally have the opinion that physicians hold a unique set of skills and knowledge that can highly impact society and save lives. Holding the physician accountable for every phone call or professional conversation could negatively impact the willingness of medical specialtists to provide others with general recommendations.

The court’s hesitation to hold consultants liable was illustrated in the case of *Bessenyei v Raiti*. The patient suffered an injury after paint thinner was injected into his thumb. Initially he was cared for by Dr. Raiti in the ED. Dr. Raiti recommended that the patient seek care at a specialized center immediately. However, the patient refused and was subsequently discharged home with antibiotics and a follow-up on the following Monday. Dr. Raiti had called Dr. Birely, a plastic surgeon, who agreed with Dr. Raiti’s plan. The patient’s condition deteriorated resulting in an amputation to the tip of his thumb. The patient sued both Dr. Raiti and Dr. Birely. The court ruled that Dr. Birely was not liable as no patient-physician relationship had been established for the following two reasons: 1) Dr. Birely was not on-call that evening, and although he agreed with Dr. Raiti, he did not provide specific advice for the patient; 2) Dr. Raiti was the immediate care provider who independently had the ability and control to either accept or reject Dr. Birely’s recommendations.^33^

A contrasting example is in *Diggs v Arizona Cardiologists, Ltd*. Dr. Valdez, a cardiologist, was determined to hold a duty of care toward Mrs. Diggs during what appeared to be an “informal consult.” Mrs. Diggs presented to the ED with chest pain, where she was originally being cared for by Dr. Johnson, an EP. Dr. Johnson thought that the most likely diagnosis was pericarditis but was also considering the diagnosis of myocardial infarction. Therefore, he sought expert advice from Dr. Valdez, who happened to be passing by the ED that day. Although he was not the on-call cardiologist, Dr. Valdez reviewed Dr. Johnson’s history, physical examination, and the patient’s ECG. He agreed with Dr. Johnson that it was most likely pericarditis. Mrs. Diggs was discharged from the ED. Three hours later, she suffered a cardiac arrest and died.

Her family brought suit against both the cardiologist and the EP. Dr. Valdez claimed that he did not have a patient-physician relationship, and thus no liability. The court determined that Dr. Valdez reviewed the specific information available on Mrs. Diggs and gave advice and recommendations in his role as an expert that impacted the care Dr. Johnson provided. The court thus held Dr. Valdes was a liable party. Although he was not the on-call cardiologist, the court deemed that a patient-physician relationship had been established as he had given advice specific to Mrs. Diggs’s condition. Additionally, the court ruled that Dr. Johnson lacked the necessary skills and knowledge to interpret the ECG as he did not have admitting privileges. The court held that Dr. Johnson had relied on the expertise of Dr. Valdez when he made his decision to discharge the patient.^35^

## CONCLUSION

The reader should now clearly understand the rationale that led to the legal outcomes in these three reported cases. The first case represents a typical scenario in which all parties were held accountable for medical negligence; each of them had patient-physician relationship and performed an affirmative act. In the second case, the court ruled that there was no affirmative act made by the consultant; therefore the consultant was not held liable. Finally, in the third case, the court clearly stated why they made their decision. The consultant made an affirmative act that impacted the care delivered in the ED.

In the three cases presented there were poor patient outcomes after interactions between EPs and consultants. Courts have defined when consultant/EP co-participation in cases results in specific liability. It is important for the EP to understand when they are solely responsible for a patient, and when consultants will share the liability. If the EP desires consultant’s shared liability, the EP should take the initiative to establish the physician-patient relationship between the consultant and the patient. The simplest way to ensure that consultants share liability is to solicit an “affirmative act.” This could be accomplished by asking the consultant to see the patient or alternatively discuss the patient’s *specific* details including history, physical examination, and test results.

### Take-home Points

1. The accuracy of identifying STEMI on ECG is less than 70%. It is critical to recognize ECG abnormalities to avoid liability and poor patient outcome.
2. Missed myocardial infarction is extremely significant as it accounts for 5% of EP malpractice claims and represents up to 20% of EP dollars paid out in those claims.
3. Aortic dissection is rare, and the most common lawsuit is for failure to diagnose.
4. The ability to intervene and improve mortality hinges upon being able to make the diagnosis accurately and timely and requires the following:
5. Consideration of AD within a differential diagnosis for an individual patient
  - Using the appropriate testing
  - Correctly interpreting the results of the testing.
  - For a consultant to be held liable, the consultant must have a patient-physician relationship that is established by an “affirmative act.” Otherwise, the EP may solely hold the liability.
6. An affirmative act is elicited by seeing the patient or providing patient-specific advice based on a specific patient’s information.
7. An affirmative act is not obtained by answering a general question that is not patient specific.
